# An Experimental Study to Evaluate the Protective Effects of *Solanum lycopersicum* Seed Essential Oil on Diabetes-Induced Testicular Injuries

**DOI:** 10.3390/medicina55080499

**Published:** 2019-08-19

**Authors:** Javid Kermani, Nader Goodarzi, Mitra Bakhtiari

**Affiliations:** 1 DVM Student, Faculty of Veterinary Medicine, Razi Universtiy, Kermanshah 6714414971, Iran; 2 Department of Basic and Pathobiological Sciences, Faculty of Veterinary Medicine, Razi Universtiy, Kermanshah 6714414971, Iran; 3 Fertility & Infertility Research Center, Kermanshah University of Medical Sciences, Kermanshah 6715847141, Iran

**Keywords:** *Solanum lycopersicum*, testis, diabetes, oxidative stress, stereology

## Abstract

*Background and Objectives*: Diabetes is a chronic metabolic disorder that can effectively influences male reproductive performance. The present study was conducted to investigate the protective effects of *Solanum lycopersicum* essential oil (SL) on diabetes-induced testicular injuries. *Materials and Methods*: Adult male rats were randomly allocated into five groups (n = 8 in each group). 1: control; 2: diabetic; 3: diabetic + 30 mg/kg of SL essential oil; 4: diabetic + 90 mg/kg of SL essential oil; 5: diabetic + 270 mg/kg of SL essential oil extract. Diabetes was induced by a single dose of streptozotocin (55 mg/kg) intraperitoneally. Testicular changes were assessed quantitatively using stereological method followed by measuring antioxidant enzymes including catalase, superoxide dismutase, and glutathione peroxidase, and the serum testosterone level. Malondialdehyde (MDA) levels and *Bcl*-2expression were also evaluated in the tissue samples. *Results*: Diabetes resulted in significant deleterious changes in the structure of testicular tissue, suppressed antioxidant enzymes and testosterone levels, and increased lipid peroxidation. The expression of *Bcl*-2 was downregulated in diabetic testis and resulted in enhanced apoptosis. Following 8 weeks of treatment with SL essential oil, there were noticeable improvements in the structural changes of testis and the restoration of antioxidant defense and testosterone levels in testicular tissue, especially at higher doses. *Conclusion*: In conclusion, these findings reveal that the essential oil of *Solanum lycopersicum* has potent antioxidant properties and can attenuate the adverse effects of diabetes on male reproduction.

## 1. Introduction

Diabetes is a chronic metabolic disorder caused by disturbances in carbohydrate, protein, and lipid metabolism, and is mainly due to the decreased levels of insulin secretion or reduced sensitivity of target tissues [1]. Diabetes exerts different morphological and functional changes on organ systems and can lead to chronic nephropathy, retinopathy, cardiomyopathy, and neurological complications [2]. Furthermore, experimental and clinical research on men and animal models has been shown that diabetes can effectively influence male reproductive system and results in reduced testosterone synthesis and secretion, impaired spermatogenesis, decreased libido and, eventually, infertility [2,3,4,5]. There is a wealth of supportive evidence that implicates oxidative stress as one of the main causes of diabetic complications. However, the role of oxidative stress in the progression of reproductive dysfunction is not fully understood [6], although it has been established that diabetes-induced oxidative stress can induce germ cells apoptosis through activating c-Jun N-terminal kinase and *Bcl*-2-associated X protein pathways. Thus, it has been supposed that apoptotic process can be inhibited by treatment with various antioxidants that enhance testicular antioxidant defense [7,8,9,10]. 

Previous studies have reported on various agents with potential antioxidant properties that could effectively result in improvements following diabetes-induced testicular damage [11,12,13,14]. Some works revealed positive effects of different essential oils on sperm and testis damage following copper [15], busulfan [16], and taxane [17] poisoning. Furthermore, some essential oils can improve semen and sperm quality under normal conditions [18,19]. *Solanum lycopersicum* is a commonly used vegetable rich in various constituents with antioxidant and anti-inflammatory properties, such as lycopene, beta-carotene, folate, flavonoids, and vitamin C [20,21,22,23]. The therapeutic or preventive effects of this plant in cancer [24,25,26], atherosclerosis [27,28], and asthma [29,30] has been confirmed. Furthermore, noticeable hypoglycemic and hypolipidemic properties of tomato have also been found [31,32]. Nevertheless, the antioxidant properties of *Solanum lycopersicum* seed essential oil have not previously been evaluated. Therefore, the present study was designed to investigate whether *Solanum lycopersicum* seed essential oil could attenuate diabetes-induced testicular injuries. 

## 2. Materials and Methods

### 2.1. Essential Oil Extraction

In this study, essential oil of SL seed extracted using a Soxhlet apparatus. Seeds of *S. lycopersicum* (500 g) were collected and dried for 7 days in shade. After grinding the dried seeds, the obtained powder was added to a distillation flask (1L) which was connected to a steam producer via a glass tube and to a condenser to collect the essential oil in a funnel tube. Components of the essential oil were purified and evaporated into hot steam, and the steam containing the essential oil was then compressed through a cooling system and essential oil was withdrawn in small and dull vials and stored in a refrigerator. The chemical constituents of the obtained essential oil were determined by gas chromatography/mass spectrometry (GC/MS).

### 2.2. Antioxidant Activity Measurement with 2,2-Diphenyl-1-Picrylhydrazyl (DPPH)

The antioxidant activity of a hydroalcoholic extract of SL essential oil was evaluated using the DPPH free radical assay. When DPPH reacts with an antioxidant compound which can donate hydrogen, it is reduced. The change in color from deep violet to light yellow is then read using a spectrophotometer. In this study, 11 different dilutions of SL essential oil were investigated. DPPH (200 μL) was dissolved in ethanol, and the DPPH solution was added to each dilution. After incubation in darkness for 30 min, its absorption was assessed at 517 nm wave lengths. Vitamin E with similar concentration was used as a positive control, and the free radical scavenging activity was calculated using the following formula:

AA% = [A0 − A1/A0] × 100,

where A0 is absorption of DPPH and A1 is the absorption of vitamin E and SL seed essential oil [33].

### 2.3. Animals and Experimental Design

Adult male rats (weighing 240–280 g, 13 weeks old) were purchased from animal house, Faculty of Veterinary Medicine, Razi University, Iran. The animals were kept under standard conditions (12 h dark/12 h light cycle, temperature 23 ± 1 °C, and humidity 50% ± 55%) and fed ad libitum. All experimental procedures were conducted according to the Ethics Committee of Razi University (approval no: 397-2-006; 14 July 2018)

After one week, diabetes was induced by intraperitoneal injection of a single dose (55 mg/kg/bw) of streptozotocin (STZ) (Sigma, St. Louis, MO, USA). At 3 days after STZ injection, blood glucose levels were monitored in 8h-fasted animals using a strip glucometer. The animals with blood glucose higher than 250 mg/dL were considered diabetic and included in the experiment. 

Animals were weighed and randomly divided into five groups, with 8 animals in each group (*n* = 8):

Group 1: served as control group, received 0.5 mL of normal saline/day orally for 8 weeks.

Group 2: served as diabetic group, received 0.5 mL of normal saline/day orally for 8 weeks.

Group 3: served as diabetic treated group, received SL essential oil at a dose of 30 mg/kg orally for 8 weeks.

Group 4: served as diabetic treated group, received SL essential oil at a dose of 90 mg/kg orally for 8 weeks.

Group 5: served as diabetic treated group, received SL essential oil at a dose of 270 mg/kg orally for 8 weeks.

### 2.4. Hormone Assay

At the end of the experiment, the animals were subjected to deep anesthesia with chloroform, and blood samples were collected directly from the heart. The blood samples were centrifuged at 1000 rpm (EBA-20, Hettich, Tuttlingen, Germany) for 10 min and the separated serum was used for measuring the testosterone level. 

Serum testosterone level was determined by enzyme-linked immunosorbent assay (ELISA) method using special rat kits (CusabioBiotech, Wuhan, China). The Testosterone ELISA kit was a colorimetric competitive enzyme immunoassay kit which provides results in 3 h. Absorbance was read at 405 nm. Ready-to-use liquid color-coded reagents are provided to reduce error. 

### 2.5. Antioxidant Enzymes Activities

The right testis of each animal was used for evaluation of antioxidant enzymes. A 2 g sample of each testis was homogenized (Ultra Turrax mechanical blender) in 50 vol of 10 mM potassium phosphate buffer (pH = 7.4) supplemented with 30 mM KCl.

The testicular homogenates were centrifuged at 13,000*g* for 15–20 min at 4 °C to obtain the enzyme fraction. The obtained supernatant was used for determination of antioxidant enzyme activity and levels of lipid peroxidation (MDA activity). Catalase (CAT) activity was measured according to the UV colorimetric method described by Aebi (1984) [34] at 240 nm using the oxidizing reaction of nitroblue tetrazolium (NBT) and H_2_O_2_ as substrate. Superoxide dismutase (SOD) activity was determined according to the colorimetric method of Martin et al. (1987) [35] at 560 nm. Glutathione peroxidase (GPx) activity was measured according to the method described by Ho et al. (1997) [36] at 340 nm. The malondialdehyde (MDA) level was measured by spectrophotometry as per the method of Placer et al. (1966) [37]. This method was based on the reaction of MDA with thiobarbituric acid (TBA), one of the aldehyde products of lipid peroxidation [37].

### 2.6. Stereological Measurements

**Figure 1:**
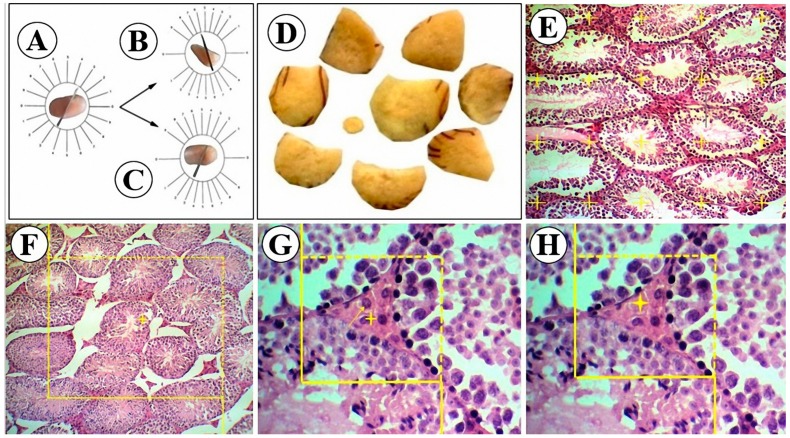
Stereological procedures for quantitative analysis. (**A**–**C**): Orientator method. (**A**) Dividing the testis into two halves at a random selected direction in the evenly divided circle. (**B**,**C**) Cutting each half in a randomly selected direction in the unevenly cosine-weighted divided circle. (**D**) A total of 8–10 slabs were collected from each testicle. (**E**) Point counting method to estimate the volume density of the desired structures. (**F**) Unbiased counting frame for estimating the length density of the seminiferous tubules. (**G**,**H**) Physical dissector method for estimating the numerical density, consists of reference plane and look-up plane, respectively. The cell that was found in the reference plane (*arrow*)and that disappeared in the look-up plane (*asterisk*) was counted.

The left testis of each animal was allocated for quantitative measurements and were immediately placed in Bouin’s solution after removing and weighing. After 48 h fixation, due to the twisty arrangement (anisotropy) of the testicular tubules, the tissue samples were subjected to orientator method to obtain isotropic uniform random (IUR) slabs [38]. A brief description of this method is presented under the Figure 1A–C. A total of 8 to 10 slabs were harvested from each testis (Figure 1D). These slabs were then processed routinely in a tissue processor set (DS2080/H, Iran) and embedded in paraffin. Tissue sections of 5 µm thickness were obtained using a rotatory microtome and stained with hematoxylin and eosin (H&E). 

#### 2.6.1. Volume Estimation

Fractional volume (volume density) of testis compartments (seminiferous tubules and interstitial tissue) were estimated using a point counting probe (Figure 1E). A point probe consisting of 25 points (+) was superimposed on the section images and the volume density of the desired structures were estimated by dividing the sum of the points hitting the desired structures over the sum of the points hitting the total section as the following formula shows:

V_v_ = ∑P_structure_/∑P_total_.

Overall, one section of each organ and 14 field of views of each section were examined. For obtaining the total volume of each structure, the volume density was multiplied by the reference volume (total volume of the testis):

V_total/structure_= V_v_ × V_reference_

#### 2.6.2. Tubule Length Estimation

The length density of seminiferous tubules was calculated using an unbiased counting frame [39]. A 10 cm × 10 cm counting frame with exclusion lines (continuous borders) and inclusion lines (dashed borders) was designed and superimposed on the live image sections (Figure 1F). The length density was calculated as follows [40]:

L_v_ = 2 × ∑Q/a(frame) × ∑frame,

where ∑Q is sum of the tubule profiles that were completely or partly inside the counting frame but only touched the inclusion lines, a(frame)is the area of the counting frame (100 cm^2^), and ∑frame is the total number of examined frames. The total length of the profiles was estimated by multiplying the length density by the reference volume. The diameter of the tubules was measured perpendicular to the long axis where the tubule was widest. An average of 100 profiles/testis.

#### 2.6.3. Germinal Epithelium Height Estimation

The height of the germinal epithelium was estimated using the following formula [39]:

H = V_v_/S_v_

in which V_v_ and S_v_ were the volume density and surface density of the germinal epithelium, respectively. The surface density of the germinal epithelium was estimated using a linear test probe.

#### 2.6.4. Number Estimation

The numerical density of Sertoli and Leydig cells was estimated using the physical dissector method [41]. In this method, two dissector probes (500 µm × 500 µm) with exclusion (left and lower) and inclusion (right and upper) borders were designed and superimposed on the images of two consecutive sections. The first section was considered as the reference plane (Figure 1G) and the second one as look-up plane (Figure 1H). According to the rules of the physical dissector method, a cell was counted if it was observed in the reference plane but not in the look-up plane, as well as did not contact the exclusion lines of the probe. At least 200 cells/testis were counted. The numerical density was estimated using

N_v_ = ∑Q/a(frame) × h × ∑P,

where ∑Q is the sum of the counted cells, a(frame) is the probe area (25 × 10^4^ µm^2^), ∑P is the total number of examined fields, and h is the dissector height (5 µm). The total number of the Leydig cells was estimated by multiplying the numerical density by the final testis volume.

#### 2.6.5. Estimation of the Sperm Tail Length

**Figure 2:**
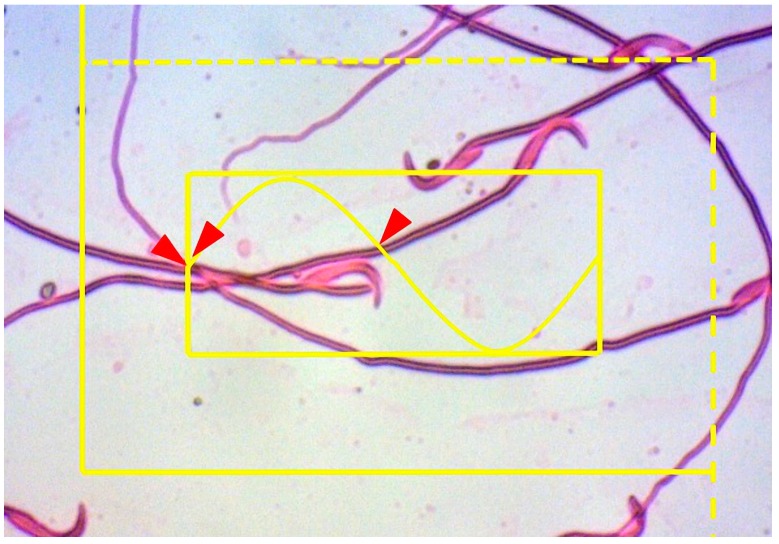
System composed of two elements (an unbiased counting frame and a basic tile with a Merz grid inside was superimposed on the image. If the sperm head lay inside the frame and did not touch the forbidden (dotted) lines, it was sampled (here, 3 sperm).

The left cauda epididymis was dissected and cut into small pieces in a petri dish containing 2 mL of phosphate-buffered saline (PBS, pH = 7.4). The suspension was then incubated at 37 °C for 15 min to disperse more spermatozoa. Afterwards, a drop of this suspension was placed on a microscopic slide and a smear was prepared and stained using theeosin-nigrosin method [42]. The slides were examined under oil immersion with 100× objective. To estimate the spermatocyte tail length, a Merz grid inside two counting frames (a large counting frame with exclusion and inclusion lines and a smaller tile inside that) was used (Figure 2). The mean sperm tail length was estimated using the following formula [40]:

∑*L* = (*π*/2). (1/*asf*) × (*a*/*l*). ∑*I L* = ∑*L*/∑*N*,

where *a*/*l* is the Merz grid constant, *asf* is the area of the basic tile divided by the area of the counting frame, ∑*I* is the summation of the intersection of the tails with the Merz grid, and ∑*N* is the total number of counted sperm in the unbiased counting frame. Overall, 100–200 sperm heads were sampled using the counting frame in each testis. 

### 2.7. Immunohistochemical Assay

The tissue sections were first dewaxed and incubated with methanol containing 3% H_2_O_2_ for 15 min at room temperature. Antigen retrieval was performed with 10 mM sodium citrate buffer (pH = 6) at 98°C for 15 min. For the next step, the sections were incubated with primary antibody (mouse monoclonal antibody against *Bcl*-2) overnight at 4 °C. After washing with PBS, the sections were incubated with biotinylated goat anti-mouse IgG as secondary antibody at room temperature for 30 min. Afterwards, the samples were exposed to 3,3-diaminobenzidine substrate as chromogen for 8 min, after which bound antibodies were revealed as brown staining. Counterstaining was performed using Harris hematoxylin.

The immunostaining intensity was estimated using a semi-quantitative score, HSCORE, method by using the following algorithm [43]:

HSCORE = Σpi (i + 1),

where i is the intensity of staining (0: no staining, 1: weak, 2: moderate, 3: strong) and pi is the percentage of stained cells for each intensity (0 to 100%). For each animal, at least 10 tubules were examined.

### 2.8. Statistical Analysis

The obtained results are expressed as means ± standard deviation (SD). The normal distribution of data was assessed by Kolmogorov–Smirnov test. Statistical analysis was conducted by using one-way ANOVA followed by Tukey’s post hoc test in SPSS software version 21 (SPSS Inc. Chicago, IL, USA) and *p* < 0.05 was considered significant.

## 3. Results

### 3.1. GC/MS Analysis of SL Seed Essential Oil

The results of GC/MS analysis are shown in Table 1. There were 11 chemical compounds in the SL seed essential oil, and 9,12-octadecadienoic acid (Z,Z)-, methyl ester, and 9-octadecenoic acid (Z)-, methyl ester, and hexadecanoic acid, methyl ester (with 49.68%, 22.02%,and 18.50%, respectively) were the most abundant compounds found in this essential oil.

### 3.2. Antioxidant Activity of SL Seed Essential Oil

**Figure 3:**
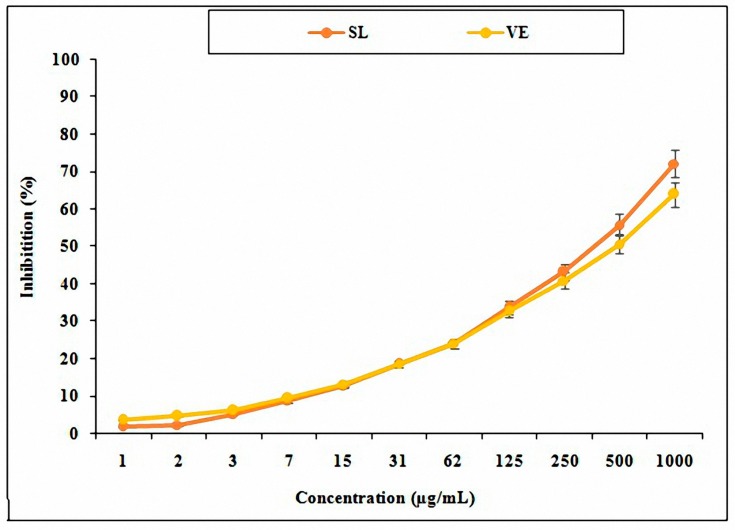
Activities of different dilutions of SL seed essential oil in comparison with vitamin E. SL: *Solanum lycopersicum*; VE: vitamin E.

The findings showed that SL seed essential oil has higher antioxidant activity when compared to the vitamin E (Figure 3).

### 3.3. Serum Testosterone Levels

**Figure 4:**
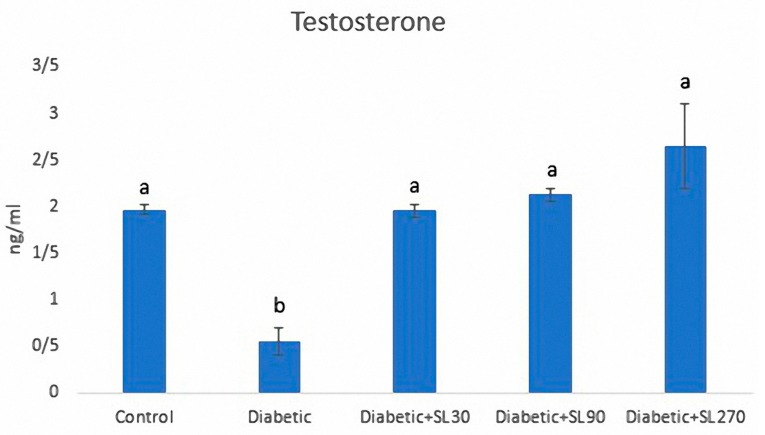
Testosterone levels in the control and experimental diabetic groups treated with 30 mg/kg (Diabetic + SL30), 90 mg/kg (Diabetic + SL90) and 270 mg/kg (Diabetic + SL270) of SL seed essential oil. The different letters indicate statistically significant differences. *p* < 0.05 (*n* = 8).

Diabetes induction resulted in a significant reduction in serum testosterone levels of diabetic rats compared to the control rats. Treatment with SL seed essential oil for 8 weeks could significantly recover the serum testosterone changes to the normal levels expected in non-diabetic rats (Figure 4).

### 3.4. Antioxidant Enzymes and MDA Activities

**Figure 5:**
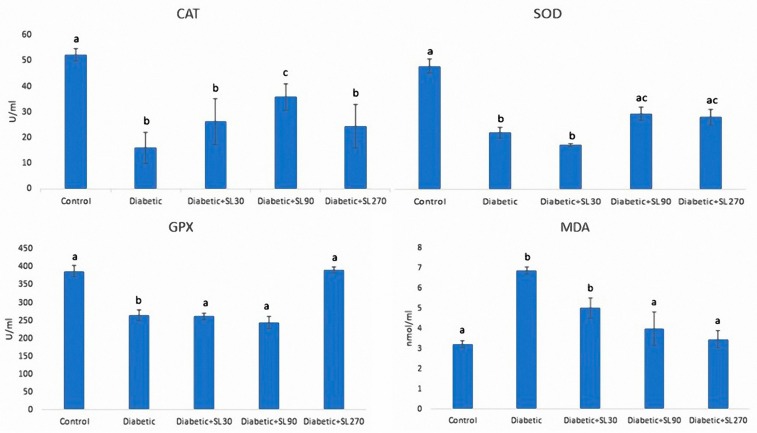
Levels of testis catalase (CAT), superoxide dismutase (SOD), glutathione peroxidase (GPx), and malondialdehyde (MDA) in the control and experimental diabetic groups treated with 30 mg/kg (Diabetic + SL30), 90 mg/kg (Diabetic + SL90) and 270 mg/kg (Diabetic + SL270) of SL seed essential oil. The different letters indicate statistically significant differences. *p* < 0.05 (*n* = 8).

As presented in Figure 5, the activities of antioxidant enzymes in testicular tissue, including CAT, SOD, and GPX, was significantly lower (*p* < 0.05) than those of the control group. The MDA activity as an indicator of lipid peroxidation also showed a significant (*p* < 0.05) increase in diabetic subjects compared to the controls. Treatment of diabetic rats with higher doses of SL seed essential oil effectively improved the activities of SOD and GPX toward normal levels. The activity of CAT was increased with dose of 90 mg/kg SL seed essential oil, albeit not to the levels in the control group. The levels of MDA in diabetic rats treated with doses of 90 and 270 mg/kg SL seed essential oil were significantly (*p* < 0.05) lower than those of the non-treated diabetic rats.

### 3.5. Testis Stereological Parameters

**Figure 6:**
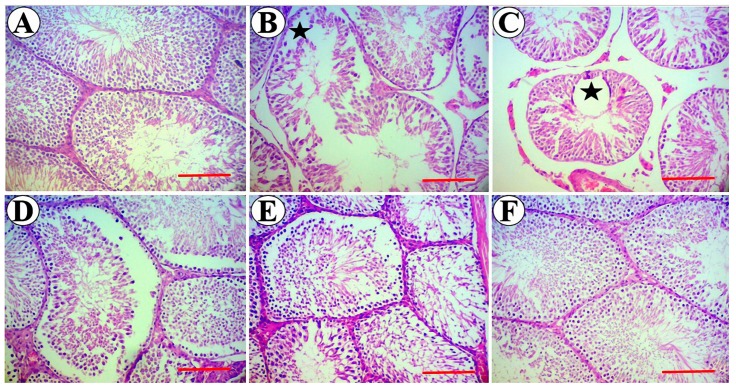
Photomicrograph of testicular tissue in the control, diabetic, and experimental groups. (**A**) Normal structure of the testis with regular arrangement in the spermatogenic cells series in the control group. (**B**,**C**) Damaged testicular tissue in the diabetic group showed degenerative changes such as disorganization and vacuolization (*asterisk*) in the spermatogenic cells series, detachment of germinal epithelium, and depletion of the interstitial connective tissue. (**D**–**F**) Testicular tissue in the treated groups received 30, 90, and 270 mg/kg of SL seed essential oil and showed improvements in diabetes-induced structural changes toward normal appearance (H&E staining, 100×, scale bar = 100 µm).

The weight and total volume of the testis showed significant (*p* < 0.05) decrease after 8 weeks of diabetes induction (Table 2 and Table 3). The tubule volumes as well as the volume and height of its germinal epithelial were significantly diminished (*p* < 0.05) in diabetic rats compared to the control group. The volume of the interstitial tissue was also significantly decreased in diabetic rats compared to the control group. The diameter of tubules and their luminal diameter were affected and significantly reduced (*p* < 0.05) in the diabetic group in comparison with the control group (Table 3). The results also showed a respective reduction in tubule lengths of diabetic rats as compared to the controls. Measurement of sperm tail lengths showed that the tail lengths were significantly lower in diabetic animals as compared to the controls (Table 4). Analysis of the cells total number revealed significant (*p* < 0.05) losses in the Leydig and Sertoli cells of diabetic rats compared to the controls (Table 5). According to the obtained results, simultaneous administration of higher doses of SL seed essential oil in diabetic subjects could notably enhance the examined structural parameters toward the normal control levels. In this regard, there were no statistically (*p* > 0.05) significant differences between the doses of 90 and 270 mg/kg of SL seed essential oil, except for sperm tail length and cell numbers, where the high dose (270 mg/kg) of SL essential oil showed better effects. Histopathological examination of tissue sections showed normal architecture of seminiferous tubules and interstitial tissue in testicles of the control group with no evidence of degenerative changes or detachment and vacuolization in the spermatogenic cell line. Induction of diabetes led to varying degrees of degenerative changes and detachment in germ cell layers which eventually resulted in reduced epithelial height and volume as well as am increase in the lumen diameters of seminiferous tubules. These detrimental changes began to disappear and the normal structure of testicles was noticeably recovered in diabetic groups treated with higher doses of SL seed essential oil (Figure 6A–F).

### 3.6. Expression of Bcl-2

**Figure 7:**
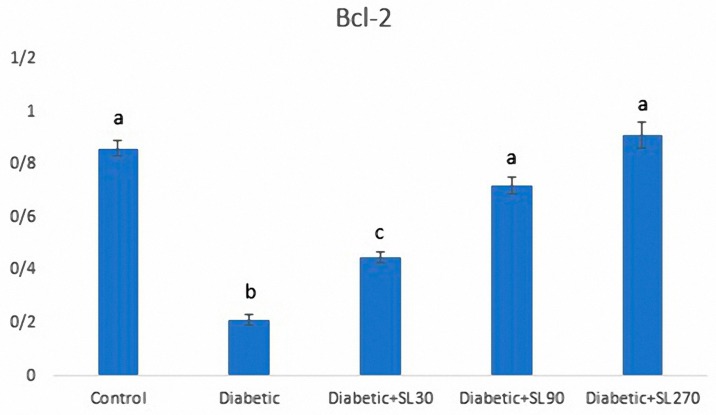
Assessments of *Bcl*-2 expression in the control and experimental diabetic groups treated with 30 mg/kg (Diabetic + SL30), 90 mg/kg (Diabetic + SL90) and 270 mg/kg (Diabetic + SL270) of SL seed essential oil. The different letters indicate statistically significant differences. *p* < 0.05 (*n* = 8).

**Figure 8:**
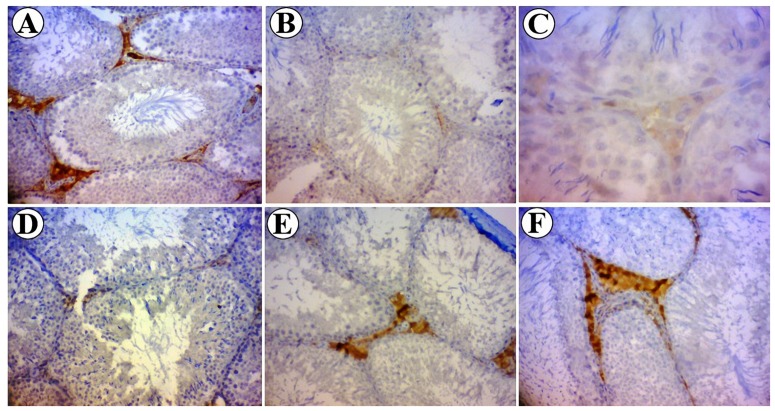
*Bcl*-2 in the testicular cross sections of control and experimental diabetic groups. (**A**) Control group showed moderate to strong immunostaining in spermatogenic cells and interstitial cells. (**B**,**C**) (at higher magnification). Diabetic groups showed weak or negative immunoreactivity pattern. (**D**) Diabetic + SL30 group, (**E**) Diabetic + SL90 group and (**F**) Diabetic + SL270 group showed moderate to strong immunostaining similar to the control group. (Magnifications: 400×).

HSCORE evaluations of *Bcl*-2 expression in seminiferous tubules and the intensity of immunoreactivity of *Bcl*-2 in different experimental groups are shown in Figure 7 and Figure 8. HSCORE assessments showed that *Bcl*-2 expression was significantly decreased in testicular cells of diabetic rats (*p* < 0.05). SL seed essential oil-treated animals showed a significant increase in the expression of *Bcl*-2, especially in Leydig cells, as compared to the diabetic animals (*p* < 0.05) (Figure 7). In the control group, Leydig cells showed strong immunostaining in their cytoplasm. Primary spermatocytes and spermatids showed moderate immunostaining (Figure 8A). In diabetic rats, Leydig cell showed weak immunoreactivity to *Blc*-2, and different types of germ cells showed negative immunostaining (Figure 8B). In SL seed essential oil-treated animals, the pattern of immunolocalization of *Bcl*-2 in seminiferous tubules was similar to the control group (Figure 8C–E).

## 4. Discussion

The present study indicated that STZ-induced diabetes in adult male rats caused testicular dysfunction and administration of *Solanum lycopersicum* seed essential oil effectively attenuated these complications due to its potent antioxidant compounds.

It is well known that STZ-induced diabetes has devastating effects on the male reproductive system. These injuries are due more to the development of diabetes, than to the toxic effects of streptozotocin or its derivatives [44]. In fact, elevation in blood glucose levels would lead to increased production of reactive oxygen species (ROS). Therefore, oxidative stress acts as a trigger, which results in decreased antioxidant defense and, ultimately, damage to the cell membrane and apoptosis in the testis.

As the obtained results show, STZ-induced diabetes can result in increased lipid peroxidation and MDA activity. As expected, the activity of antioxidant enzymes, including CAT, SOD, and GPx, were greatly diminished in testicular tissue. These defects were associated with apoptosis, which was confirmed by the decrease in Leydig and Sertoli cell number as well as the downregulation of the anti-apoptotic gene, *Bcl*-2. Furthermore, a reduction in germinal epithelial height and volume, which were observed following STZ-induced diabetes, can be attributed to progressive apoptosis in the spermatogenic cell series. Evidently, germinal epithelia constitutes the most part of the seminiferous tubules, and the seminiferous tubules organize the main compartment of testicles. Therefore, consistent with our data, it can be expected that decreasing thickness of the germinal epithelium would be accompanied by a reduction in the volume of tubules and, consequently, also in the volume of the testis [8,13]. These changes, including cellular apoptosis and testicular atrophy, can result in impaired spermatogenesis and eventual infertility. The obtained results for sperm tail length revealed shorter sperm tails in diabetic animals as compared to the controls. This finding can be indicative of impaired spermatogenesis. Our results suggest that due to its inherent biological compounds, seed essential oil have considerable antioxidant potential and could prevent the abovementioned oxidative stress-induced testicular damage following diabetes. In the present study, it was found that seed essential oil, especially at higher examined doses, could effectively enhance the activity of antioxidant enzymes in testicular tissue and with consequent improvements regarding apoptosis and testicular atrophy, which is reflected in the increase in germinal epithelial height and production of healthy sperms with normal tail length.

Taking another point of view, testicular atrophy or hypogonadism in diabetes can be attributed to the impaired synthesis or secretion of testosterone hormone [45]. Several mechanisms were suggested for low testosterone levels in diabetic individuals. At first, decreasing glucose uptake by anterior hypophysis results in reduced secretion of LH and FSH. Furthermore, insulin is a stimulus for secretion of gonadotropins. Some authors revealed that reduced levels of gonadotropins in diabetic subjects might be due to the insufficient secretion of GnRH from the hypothalamus or an impaired hypophyseal response to GnRH [46]. Diabetes-induced alterations in the proliferation, differentiation, and function of Leydig cells should also be considered [47]. The present results likewise showed lower testosterone levels in diabetic rats. Fortunately, seed essential oil could significantly restore the testosterone levels in diabetic animals as compared to the non-treated diabetic rats. In this study, the Leydig cells of diabetic animals were assessed both quantitatively using physical dissector and qualitatively for *Blc*-2 expression. Earlier studies have shown that hyperglycemia increases the expression of *Bax* protein, as a pre-apoptotic gene, and causes apoptosis in PC1 cells [48]. In the testis of diabetic rats, apoptosis is accompanied by downregulation of *Bcl*-2 gene expression [49]. This study showed that treatment of diabetic rats with SL seed essential oil prevented apoptosis through upregulation the of *Bcl*-2 protein expression.

The results of the present work indicated that SL seed essential oil could effectively improve testicular injuries following STZ-induced diabetes. Different earlier studies introduced several compounds such as ferulic acid [50], melatonin [13], and quercetin [51], which have protective effects on post-diabetes testicular damage. The authors concluded that these beneficial effects are mainly due to their antioxidant activities. It has been revealed that aqueous extracts of *S. lycopersicum* inhibit the expression of TNF-α and IL-1β in LPS-stimulated macrophages through the inhibition of NF-κB [52]. It is well known that TNF-α promotes insulin resistance by inhibiting IRS1 (insulin receptor substrate 1) [53] and IL-1β causes apoptosis in β-pancreatic cells, thereby diminishing insulin production [54,55].

In the present study, GC/MS analysis revealed that 9,12-octadecadienoic acid (Z,Z)-, methyl ester, and 9-octadecenoic acid (Z)-, methyl ester, and hexadecanoic acid, methyl esterat 49.68%, 22.02%,and 18.50%, respectively, were the most abundant compounds found in SL seed essential oil. 9,12-Octadecadienoic acid, an omega-6 fatty acid also known as linoleic acid, is a precursor in the synthesis of arachidonic acid and other polyunsaturated fatty acids [56]. The antioxidant potential of these compounds was observed in previous studies [57,58,59,60].

In the present study, besides the stereological and quantitative assessment, testicular tissue was evaluated qualitatively, and testicular dysfunction was confirmed by disorganization and vacuolization in the architecture of seminiferous tubules and detachment of spermatogenic cell series. All of these histopathological alterations were normalized upon treatment with SL seed essential oil. These effects can be also rationalized by the antioxidant properties of SL seed essential oil.

## 5. Conclusions

The findings of the present study indicated that STZ-induced diabetes results in profound structural and biochemical alterations in the testis of diabetic rats, and that higher doses of SL seed essential oil, with antioxidant properties, can improve diabetes-induced oxidative stress and the structural changes in the testis by strengthening the testicular antioxidant defense system.

## Figures and Tables

**Table 1:** Chemical compounds in *Solanum lycopersicum* (SL) seed essential oil determined by GC/MS analysis.

Compounds	RT (min)	Percentage of the Total Chromatogram Area
2H-Oxireno [3,4] cyclopenta [1,2-c] furan-2-one, 1a,1b,4,4a,5,5a-hexahydro-4-(dimethoxymethyl)-, (1bR,1a-cis,4-trans,4a-cis,5a-cis)-	6.057	0.305%
Nonanal dimethyl acetal	6.123	0.264%
9-Hexadecanoic acid, methyl ester, (Z)-	10.999	0.301%
adecanoic acid, methyl ester	11.179	18.509%
n-Hexadecanoic acid	11.476	0.722%
9,12-Octadecadienoic acid (Z,Z)-, methyl ester	12.696	49.68%
9-Octadecenoic acid (Z)-, methyl ester	12.735	22.021%
Octadecanoic acid, methyl ester	12.898	6.474%
9,12-Octadecadienoic acid (Z,Z)-	12.983	0.98%
Octadecanoic acid	13.195	0.37%
Hexadecanoic acid, 15-methyl-, methyl ester	14.558	0.374%
Total		100

RT: Retention Time.

**Table 2:** The initial and final body weight and testis weight in the control and experimental groups.

Groups	Control	Diabetic	Diabetic + SL30	Diabetic+SL90	Diabetic+SL270
Initial body weight (g)	265 ± 12	252 ± 15	253 ± 22	244 ± 14	238 ± 18
Final body weight (g)	262 ± 51 ^a^	173 ± 5 ^b^	221 ± 40 ^a^	220 ± 19 ^a^	190 ± 3 ^b^
Testis weight (g)	1.4 ± 0.15 ^a^	0.85 ± 0.1 ^b^	0.97 ± 0.09 ^b^	1.42 ± 0.04 ^a^	1.56 ± 0.05 ^a^

Data were expressed as mean ± SD (*n* = 8). Different superscripts in the same row indicate significant difference (*p* < 0.05). Diabetic + SL30 treated with 30 mg/kg, Diabetic + SL90 treated with 90 mg/kg and Diabetic + SL270 treated with 270 mg/kg of SL seed essential oil.

**Table 3:** Total volume (mm^3^) of testis, seminiferous tubules, interstitial tissue, epithelium, tubule diameter (µm), and height of the germinal epithelium (µm) in the control and experimental groups.

Groups	Control	Diabetic	Diabetic + SL30	Diabetic+SL90	Diabetic+SL270
Testis volume	1150.6 ± 110 ^a^	712.2 ± 34 ^b^	815.5 ± 12 ^b^	1108.4 ± 115 ^a^	1222 ± 108 ^a^
Tubule volume	948.3 ± 84 ^a^	605.1 ± 108 ^b^	732 ± 24 ^b^	918.5 ± 121 ^a^	1064.8 ± 119 ^a^
Interstitial tissue volume	126.2 ± 44 ^a^	98.8 ± 35 ^b^	81.2 ± 14 ^b^	117.9 ± 24 ^a^	131.8 ± 28 ^a^
Epithelial height	118.7 ± 12 ^a^	77.3 ± 9 ^b^	81.3 ± 24 ^b^	125.5 ± 32 ^a^	118.5 ± 16.5 ^a^
Tubule diameter	305.5 ± 31 ^a^	209.2 ± 12 ^b^	226.1 ± 8 ^b^	281.9 ± 34 ^a^	289 ± 41 ^a^

Data were expressed as mean ± SD (*n* = 8). Different superscripts in the same row indicate significant difference (*p* ≤ 0.05). Diabetic + SL30 treated with 30 mg/kg, Diabetic + SL90 treated with 90 mg/kg and Diabetic + SL270 treated with 270 mg/kg of SL seed essential oil.

**Table 4:** Length of seminiferous tubules (m) and sperm tail (µm)in the control and experimental groups.

Groups	Control	Diabetic	Diabetic + SL30	Diabetic+SL90	Diabetic+SL270
Tubule length	22.2 ± 3.1 ^a^	13.5 ± 0.8 ^b^	12.1 ± 1.5 ^b^	18.5 ± 7.1 ^a^	19.2 ± 4.7 ^a^
Sperm tail length	30.4 ± 4.8 ^a^	18.2 ± 7.8 ^b^	20.5 ± 4.2 ^b^	19.4 ± 5.8 ^b^	25.6 ± 2.9 ^a^

Data were expressed as mean ± SD (*n* = 8). Different superscripts in the same row indicate significant difference (*p* ≤ 0.05). Diabetic + SL30 treated with 30 mg/kg, Diabetic + SL90 treated with 90 mg/kg and Diabetic + SL270 treated with 270 mg/kg of SL seed essential oil.

**Table 5:** Numbers (×10^6^) of Leydig cells and Sertoli cells in the control and experimental groups.

Groups	Control	Diabetic	Diabetic + SL30	Diabetic+SL90	Diabetic+SL270
Leydig cells	6.2 ± 0.8 ^a^	4.2 ± 0.25 ^b^	4.8 ± 0.5 ^b^	4.6 ± 0.21 ^b^	5.9 ± 0.92 ^a^
Sertoli cells	13.7 ± 2.1 ^a^	7.5 ± 1.8 ^b^	8.2 ± 2.1 ^b^	7.6 ± 0.41 ^b^	15.3 ± 0.55 ^a^

Data were expressed as mean ± SD (*n* = 8). Different superscripts in the same row indicate significant difference (*p* ≤ 0.05). Diabetic + SL30 treated with 30 mg/kg, Diabetic + SL90 treated with 90 mg/kg and Diabetic + SL270 treated with 270 mg/kg of SL seed essential oil.
